# February

**Published:** 1890-02-01

**Authors:** 


### FEBRUARY.

The month of February is young in comparison with the majority of the other months, and has experienced vicissitudes of fortune. It had no place in the Romulian kalendar, which consisted of ten months only. Numa added January and February, the latter being placed at the end of the year. But afterwards, 452 B.C., February was placed after January. Long before the exact length of the year was determined, it was perceived that the revolution of the moon is accomplished in 29½ days. Twelve lunations, therefore, form a period of 354 days, which differs by about 11¼ days from the solar year. It was Julius Cæsar who endeavoured to set time right by first ordering two extraordinary or supplementary months, which gave him a fair start in accordance with the seasons, and then decreeing that every fourth year should have 366 days. But this computation proved too long by eleven minutes, fourteen seconds yearly. In the course of a few centuries, the equinox sensibly retrograded towards the beginning of the year. When the Julian kalendar was introduced, 46 B.C., the equinox fell on the 25th of March. At the Council of Nice, in 1582, it had retrograded to the 11th, so Pope Gregory XIII. tried his hand at tinkering the time. It was not, however, till 1752 that the Gregorian style was adopted in Great Britain, and then the 3rd of the month of September had to be called the 14th. By a strange misnomer we call it leap year when February's days are 29.

February is said to have taken its name from the Latin word signifying to purify. But it was probably so called because the addition of February was intended to set matters right as regards time and season. With the Romans the month of February was sacred to Mars, the Phallic god, or Februa, his wife, who forsook Hephestus, the god of fertilising fire. So some consider the name derived from the lady. The Romans worshipped with fire and lights, and the Christians, while turning from the Phallic god, retained the symbols under the authority of Pope Sergius. This pope, seeing how determined his Christian flock were to use lights, ordered them to come to church and offer up candles. The chief festivals of February are indeed those connected with candles, fire, and purification. Thus the 2nd of February is known as the purification of the Virgin Mary, called in the North of England "the wives feast day." On Candlemas Day, according to "the old foarme of ye church of England, the bearynge of candels is done in the memorie of Christe ye spirituall lighte." This practice is traceable back to the time of Pope Sergius. It is not, however, so easy to trace back the reasons why weather prognostications were made on Candlemas Day. But we have the old Scotch couplet—

"If Candlemas Day is fair and clear,
There'll be two winters in the year."

And we also have the old saying "On Candlemas Day throw candle and candlestick away." Which was all very well when people went to bed with the sun, but would not suit these days when so many often go to bed when the sun rises. The 3rd of February is St. Blaize's Day. St. Blaize appears to have been an Armenian bishop, the reputed inventor of the art of wool-combing The story goes that when St. Blaize was scourged seven women anointed themselves with the blood, whereupon their flesh was combed with iron combs, and from the wounds so made there ran nothing but milk. St. Blaize was formerly regarded as the patron saint against bones sticking in the throat. February 14th, or St. Valentine's Day, is a more important festival. This appears to have been instituted by the Christian Church. For with a faint breath of returning spring, or, in our climate, the expectation of returning spring, "all nature seems in love." Nature's command is given at this time to all bird and animal life to seek out mates. It would seem as if priests of various cults had stepped forward and invited, under cover of one of the saints, the youthful to make overtures to the opposite sex. The custom, however, of sending valentines is now dying away. St. Valentine was formerly regarded as the patron saint against nervous diseases, especially epilepsy. Next we have "collops" or Shrove Monday. Some authorities regard this as originating from our ancestors taking their leave of collops, or of salt, dried, or hung flesh, for fresh meat, which they seldom tasted in the depth of winter. Others regard the change of diet as a fit preparation for the Lenten fast; Shrove Tuesday pancakes being an additional strengthening preparation. Viewed in a health aspect, February is not a salubrious period. The old phrase, "February fill ditch," is descriptive of the time, and the zodiacal sign of the fishes is very appropriate. Rain and drizzle, drizzle and rain, are too frequent characteristics of the period. And if a fine day perchance occurs, and one is tempted to leave wrappings at home, the consequence often is a cold or something worse. Although the days are lengthening and the sun is gaining a little power, the mean temperature does not much rise, and the variations are great. Towards the end of the month we may expect a pretaste of March winds. Falls of snow are also not infrequent, and although we may expect the snow to melt tolerably soon, still, notwithstanding the delusion of St. Valentine's Day, we cannot safely make much difference in our way of living and clothing from that which we adopted in the depth of winter. The temperature in February has varied in the day time from upwards of 56 to 18, and, although this does not often occur, the changes which do present are very trying to the aged and delicate. Still there is a much greater amount of sunshine in February than in January. Let us hope that the returning power of the sun will dissipate the influenza germs, and that "the epidemic constitution of the air" will be blown away by the gales we may soon expect. Hitherto we have had a "mild winter," and well-housed and warm-clothed people are saying that we do not get the "old-fashioned frosty winter now"; some endeavouring to explain the reason by a theory of the earth turning on its axis. But the seasons were always variable. In 1824, orchards blossomed in the month of March, and in 1585 corn was in ear at Easter; 1832 and 1866 were exceptionally mild winters. On the other hand, 1826 was one of the severest winters known, and so on might be enumerated other dates proving that the seasons were always erratic. Notwithstanding the old saying, that "a mild winter makes a fat churchyard," there is ample proof that a severe winter swells the bills of mortality most, and it is proverbially fatal to elderly people. The poor who have not means equivalent with the present high price of coal, will certainly rejoice that the necessity for the consumption of fuel has been lessened by the mildness of the season.

